# The Effects of Tualang Honey on Bone Metabolism of Postmenopausal Women

**DOI:** 10.1155/2012/938574

**Published:** 2012-08-29

**Authors:** Nadia Mohd Effendy, Norazlina Mohamed, Norliza Muhammad, Isa Naina Mohamad, Ahmad Nazrun Shuid

**Affiliations:** Department of Pharmacology, Faculty of Medicine, The National University of Malaysia, Kuala Lumpur Campus, 50300 Kuala Lumpur, Malaysia

## Abstract

Osteoporosis which is characterized by low bone mass and microarchitectural deterioration with a consequent increase in bone fragility can be associated with various stimuli such as oxidative stress and inflammation. Postmenopausal women are more prone to osteoporosis due to reduction in estrogen which may further lead to elevation of oxidative stress and lipid accumulation which will promote osteoblasts apoptosis. Proinflammatory cytokines are elevated following estrogen deficiency. These cytokines are important determinants of osteoclasts differentiation and its bone resorption activity. The main treatment for postmenopausal osteoporosis is estrogen replacement therapy (ERT). Despite its effectiveness, ERT, however, can cause many adverse effects. Therefore, alternative treatment that is rich in antioxidant and can exert an anti-inflammatory effect can be given to replace the conventional ERT. Tualang honey is one of the best options available as it contains antioxidant as well as exerting anti-inflammatory effect which can act as a free radical scavenger, reducing the oxidative stress level as well as inhibiting proinflammatory cytokine. This will result in survival of osteoblasts, reduced osteoclastogenic activity, and consequently, reduce bone loss. Hence, Tualang honey can be used as an alternative treatment of postmenopausal osteoporosis with minimal side effects.

## 1. Introduction

Natural product has been used for medicinal purposes long before recorded history. Wide arrays of natural products used to treat a variety of ailments are also known as traditional medicine which is defined by the World Health Organization (WHO) as the sum total of the knowledge, skills, and practices based upon the theories, beliefs, and experiences indigenous to different cultures, whether explicable or not, used in the maintenance of health as well as in the prevention, diagnosis, improvement, or treatment of physical and mental illness [1]. Recently, it has been estimated that 80% of worldwide population rely on traditional medicines in order to meet their health care needs as an alternative to the conventional medicine [2]. Alternative medicine was first discovered as early as 5000 B.C in the Middle East, followed by the development of Ayurvedic, Chinese and Western medicine. Acceleration of the use of alternative treatment nowadays is due to rise in prevalence of chronic diseases, increasing costs of medical care and public concern on the rising adverse effects of conventional medicine.

Prevalence of chronic diseases is now increasing and contributed the most in medical care spending [3]. One of the serious diseases that is now becoming an important socioeconomic burden in many countries is osteoporosis. Osteoporosis is a systemic skeletal disease characterized by low bone mass and microarchitectural deterioration with a consequent increase in bone fragility with susceptibility to fracture [4]. According to National Osteoporosis Foundation, osteoporosis has become a major public health threat. The amount of medical care expenditure is rising tremendously due to increased elderly population as a result of increased longevity [5, 6]. Main factor causing osteoporosis are age-related decline in level of sex hormones (e.g., postmenopausal women), and secondary osteoporosis that can be due to diseases such as hypogonadism, hyperthyroidism, and gastrointestinal malabsorption syndromes [7]. Prolonged used of medications such as steroid prednisone, barbiturates, thyroxine and diabetic medications, excessive alcohol consumption, and smoking can contribute to bone loss as well [8–10]. Women are prone to tremendous estrogen reduction after menopause which will result in bone loss. This explains the fact that about 80% of the economic burden of osteoporosis has been attributed to its occurrence in women, especially in postmenopausal women [11]. Men, on the other hand, have a greater total bone mass than women, hence they are less likely to develop osteoporosis compared to women [12, 13].

To date, the main treatment of osteoporosis is hormone replacement therapy (HRT) and bisphosphonate. HRT, which contains estrogen has been shown to reduce hip fractures [14], wrist, vertebral and all nonvertebral fractures [15, 16]. Although HRT is known to be very effective in increasing sex hormone level and improving bone mass, it can also produce many adverse effects, particularly breast cancer [17]. Estrogen can enhance the rate of cell proliferation in glandular tissue of breast and could potentially act both in initiation and promotion of breast cancer [18]. A study by Collaborative Group for Hormonal Factors in Breast Cancer has found out that women who had used HRT for longer than 5 years, the relative risk of breast cancer was 1.35 [19]. Other than that, prolonged use of HRT can also result in cardiac infarction, stroke, and pulmonary emboli [20]. Route of administration of HRT can be via oral, transdermal, patches, subcutaneous, and intramuscular injection. The most widely used method of administration is via intramuscular injection due to its rapid absorption. However, this method can be very painful. Other than HRT, bisphosphonates such as alendronate and risedronate are now widely used and have been approved to be used not only for treatment but also as prevention of osteoporosis. Alendronate and risedronate are able to inhibit osteoclast-mediated bone resorption and reduce risk of fracture effectively. However, these treatments may contribute to adverse effects such as abdominal pain, joint and muscle pain, constipation [21], hypocalcemia, and osteonecrosis of the jaw bones [22].

Regardless the effectiveness of current available treatments of osteoporosis, alternative natural medicines are needed to treat this disease with minimal side effects. Honey is documented as one of the most ancient traditional remedies in history [23]. The first written reference to honey was a Sumerian tablet writing dating back to 2100–2000 B.C which mentions honey's use as a drug and ointment [24]. Since then it has become useful for both nutritional and medical purposes [25]. Honey has been of proven value in accelerating wound healing, as well as treating ulcers and skin infection [26, 27]. It has also shown to be an effective antioxidant and anti-inflammatory agent [28, 29]. Honey consists of primarily sugars such as monosaccharides, disaccharides, oligosaccharides, and polysaccharides [30] as well as enzymes such as glucose oxidase, diastase, invertase, catalase, and peroxidase [31]. Other chemical contents of honey are organic acids, ascorbic acids, vitamins, amino acids, proteins, flavonoids and phenolic acids [32, 33]. These chemical constituents of honey made it beneficial in human health.

To date, there has been a resurgence in the use of honey in treating a wide array of diseases which are not only limited to wound healing. Malaysia is one of the countries in Asia that is well known for its varieties of honeys such as Tualang, Gelam, and Belimbing honey. Tualang honey is found on Asia's largest tree, Tualang tree or *Koompassia excelsa*. It has been used widely by the local community in treating wound, as beauty products, antiageing products, and health supplements [34]. Since the last few years, Tualang honey has been used widely by the researchers in order to discover its hidden potential values. The major components of Tualang honey are furfural derivatives such as 5-(hydroxymethyl)-furfural, furfural 2-furyl methyl ketone, 5-methyl furfural, and fatty acids such as palmitic acid, ethyl linoleate, and ethyl oleate [35]. Tualang honey is known for its antimicrobial, antiparasitic, antioxidant, and anti-inflammatory effects which could be due to its chemical content that is high in antioxidative properties such as flavonoids and phenolic acids [36]. Recently, Tualang honey has been studied for its effect on bone density and a positive result was exhibited. This could make it a good potential as an alternative treatment to osteoporosis, replacing the conventional treatment. This paper focuses on the mechanisms of Tualang honey on bone density and its antiosteoporotic values particularly in postmenopausal osteoporosis women.

## 2. Oxidative Stress in Postmenopausal Osteoporosis and Antioxidative Role of Tualang Honey

Bone is continually remodelled throughout life where bone resorption activity by osteoclasts is always followed by bone formation by osteoblasts [37]. Osteoporosis occurs when this bone remodelling cycle is impaired. Hence, any factors that can impair the rate of bone remodelling will contribute to bone loss such as in postmenopausal women where estrogen reduction is the main factor. Estrogen deficiency following menopause can lead to bone loss via direct effects of estrogen on osteoclasts [38] and upregulation of osteoclastogenesis. Upregulation of osteoclastogenesis occurs via activation of receptor activator of nuclear factor kappa-B ligand (RANKL) [39] and diminishes production of osteoprotegerin (OPG), which function as osteoclastogenesis inhibitory factor [40]. Postmenopausal osteoporosis can be associated with oxidative stress which results from a disturbance in the balance between free radicals production and antioxidant protective activity. Estrogen can be considered as an antioxidant as it was found to exhibit antioxidant protection of lipoproteins in the aqueous system and was shown to increase the expression of glutathione peroxidase (GPx) in osteoclasts [41]. When body is subjected to high level of oxidative stress following estrogen reduction, lipid accumulation will occur. This will promote osteoblast apoptosis and simultaneously upregulating reactive oxygen species (ROS) production, particularly hydrogen peroxide (H_2_O_2_) and superoxide anion [42–44]. ROS may increase bone resorption through activation of NF-*κ*B which plays an important role in osteoclastogenesis. ROS can also promote osteoclast resorption activity directly or by mimicking receptor activator of NF-*κ*B (RANK) signalling which results in osteoclast differentiation [45]. Some *in vitro* and animal studies have reported that oxidative stress decreases the level of bone formation by modulating the differentiation and survival of osteoblasts [46, 47]. Postmenopausal women are not only subjected to high level of free radicals and oxidative stress, but in previous studies it was also found that they exhibited higher erythrocyte nitric oxide (NO) levels compared to nonporotic women [48]. NO is able to enhance the ability of cytokines to stimulate osteoclast activity [49] and potentiates the inhibitory effects of those cytokines on osteoblast growth [50, 51].

Ovariectomized rat is often used in postmenopausal women research as a model for osteoporosis which exhibits a progressive loss of bone matrix through a process that is similar to what occurs during postmenopausal osteoporosis [52]. This include increased rate of bone turnover with resorption exceeding formation, greater loss of cancellous than cortical bone, and decreased intestinal absorption of calcium [53]. The bone loss following ovariectomy can be due to oxidative stress. In a study by Muthusami et al. (2005), there was a significant decrease in the levels of antioxidant enzymes; superoxide dismutase (SOD), glutathione-s-transferases (GST) and GPx in the femur of ovariectomized rats. Hydrogen peroxide and lipid peroxidation were found to increase. These results have shown that oxidative stress was induced in ovariectomized rats which probably is the major factor behind the bone loss in these animals [54]. The ability of cells to scavenge the harmful ROS is mainly dependent upon the efficacy of the antioxidant defense system which comprises of enzymatic antioxidants such as SOD, GPx and catalase (CAT) and nonenzymatic antioxidants such as glutathione (GSH), vitamin C and E [55, 56]. Hence, supplementation with antioxidant is required to combat the oxidative stress and subsequently preventing bone loss. Based on recent published studies, amongst the natural products that are able to prevent osteoporosis are vitamin E tocotrienol and *Labisia pumila.* Both of these natural products possess antioxidative effects [57, 58]. According to Nazrun et al. (2011) tocotrienol exert a potent antioxidative property which is able to suppress bone resorbing cytokines and thus preventing osteoporosis.

Over the past few years, a resurgence of interest in the ability of honey as an antioxidant followed by its protective effects on bone has occurred. Tualang honey has been shown to exhibit good antioxidant and antiradical activities [59, 60]. Study performed by Zaid et al. (2010) has shown that daily consumption of Tualang honey for two weeks in female ovariectomized rats was able to promote an increase in bone density [61]. This positive effect on bone of ovariectomized rats is probably due to the antioxidants found in honey such as flavonoids and phenolic acids [62, 63]. The main phenolic and flavonoid compounds in Tualang honey include kaempferol, quercetin, ellagic acid, gallic acid, hesperetin, and catechin [64].

A study on the antioxidative compounds of Tualang honey has found that Tualang honey had the highest total phenolic and protein content compared to other types of honey; Gelam, Indian forest and Pineapple honey. Among this group of honeys, Tualang honey also had the highest ascorbic acid content which may be responsible for the elevated scavenging of the ROS. In contrast to those types of honey mentioned, Tualang honey had also shown the highest DPPH radical scavenging activity, suggesting that it may contain the most effective free-radical scavenging compounds [60]. These findings have shown that Tualang honey is a good source of antioxidant that is able to scavenge free radicals, resulting in reduced bone resorption activity by osteoclasts which subsequently maintaining the bone health.

Previous studies reported that flavonoids mainly quercetin and kaempferol exert a potent inhibitory effect on osteoclastic bone resorption and apoptosis in a rabbit long bone osteoclast model [65]. They are also involved in inhibition of NF-*κ*B and activator protein (AP-1), a transcription factor highly related to osteoclastic differentiation [66]. There were some investigations suggesting that quercetin plays an important role in bone loss inhibition by affecting osteoclastogenesis and accelerating TNF-*α*-induced osteoblast growth inhibition and apoptosis [67]. Flavonoids may also inhibit RANKL-induced formation of multinucleated osteoclasts and expression of osteoclastic differentiation markers; RANK and osteocalcin receptor [68]. Flavonoids have been shown to inhibit production of nitric oxide and expression of inducible nitric oxide synthase (iNOS) [69] which will result in inhibition of osteoclast activity. These protective mechanisms of flavonoids on bone strongly indicate that it can be considered as protective agent against bone loss.

## 3. Anti-Inflammatory Effects of Tualang Honey

Osteoporosis is more prevalent in inflammatory conditions such as rheumatoid arthritis, systemic lupus erythematosus (SLE), haematological diseases, inflammatory bowel disease and other inflammatory diseases when compared to the healthy population [70]. Proinflammatory cytokines such as tumor necrosis factor (TNF)-*α*, interleukin (IL)-1, IL-6, IL-7, IL-11, IL-15 and IL-17 are elevated in these conditions [71]. Elevation of these cytokines will then result in increase production of prostaglandin E_2_ (PGE_2_), an inflammatory mediator which consequently stimulates osteoclastic activity [72]. Therefore, osteoporosis can be strongly associated with inflammation. Activated osteoclasts are usually found in the presence of accessory cells including stromal cells, cells in osteoclast lineage and cells involved in the inflammatory responses. These cells posses the ability to express proinflammatory cytokines. Thse proinflammatory cytokines has been shown capable of stimulating osteoclastic bone resorption [73].

There is evidence from previous studies to suggest that postmenopausal bone loss may be linked to activation of osteoclasts by proinflammatory cytokines [74–76]. Estrogen is able to suppress production of these cytokines and stimulate production of OPG [77, 78]. Thus, estrogen withdrawal following menopause will result in an increase in these cytokines and simultaneously lead to downregulation of OPG, resulting in local inflammation in the bone. Ovariectomy in rats was accompanied by an increase in production of TNF-*α* and IL-1 [79]. This may influence osteoclastogenesis by stimulating self-renewal and inhibiting the apoptosis of osteoclast progenitors [80]. They also support osteoclast formation and activation mediated by RANKL and macrophage colony-stimulating factor (M-CSF). These studies have shown that postmenopausal osteoporosis can be associated with inflammation. 

Honey does not only exhibit antioxidative effects but it also possesses anti-inflammatory effects. Previous studies have shown that the level of PGE_2_ was reduced after the ingestion of honey [81]. Recent study has also shown that honey was able to exert anti-inflammatory effect via inhibition of nitric oxide [82]. Consumption of Tualang honey for two weeks in female ovariectomized rats was able to increase bone density. Tibial bones of the ovariectomized rats treated with Tualang honey were comparable to those of control nonovariectomized rats [61]. This result can strongly suggests that Tualang honey was able to restore osteoporotic bone similar to the nonosteoporotic bone.

**Figure 1:**
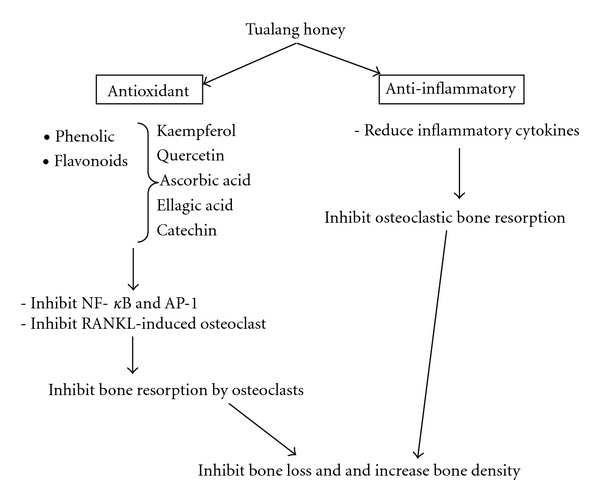
Schematic diagram of antioxidative and anti-inflammatory actions of Tualang honey on bone.

Another study performed on the effects of Tualang honey on postmenopausal women showed that daily intake of Tualang honey at 20 mg/day for four months was found to be safe and exerted the same effect on bone densitometry when compared to hormone replacement therapy [83]. Based on this result, it was shown that Tualang honey was able to produce an effective result as HRT and can be used as anti-osteoporotic agent. These positive effects of Tualang honey on bone are probably due to its anti-inflammatory property, apart from the antioxidative property mentioned. Other than that, it was found out that its anti-osteoporotic property could probably due to the presence of calcium [84] and gluconic acid. Gluconic acid could enhance calcium absorption in the bone which consequently maintaining bone mass and prevent osteoporosis [85]. These results proved that Tualang honey has the potential to be used as an alternative treatment in treating postmenopausal osteoporosis. The antioxidative and anti-inflammatory actions of Tualang honey are summarized in Figure 1. 

From nutrition point of view, Tualang honey is a sugar. For sweeteners, a maximum of 40 to 50 g per day is generally accepted. However, the recommended dose is about 20 g daily [86]. This is the reason why majority of the researches were done with the dose of 20 g daily such seen in study of the effects of Tualang honey on postmenopausal women [83]. A previous study on the hepatoprotective effect of Tualang honey supplementation in streptozotocin-induced diabetic rat has shown that diabetic rats supplemented with Tualang honey had a reduced blood glucose concentration compared to the control diabetic rats group [87]. This result suggests that Tualang honey is safe to be consumed by postmenopausal diabetic women. Since Tualang honey is a food supplement, not many studies have been done on its drug interaction. Previous study has provided evidence for thein vitroinhibition of cytochrome P450 2C8 (CYP2C8) activity by Tualang honey. It revealed that this honey, through this inhibition, may have the potential to cause *in vivo* drug-food interaction with drugs metabolized by CYP2C8 [35] such as thiazolidinediones, a class of antidiabetic drug and amodiaquine, an antimalarial agent [88]. More clinical studies should be performed in order to study the safety of Tualang honey on diabetic patients and its interaction with other drug hence, providing a more accurate proof of its safety profile.

Based on the positive effects on bone density, Tualang honey has the potential to be used as an alternative treatment for postmenopausal osteoporosis due to its anti-oxidative and anti-inflammatory properties against bone loss. As Tualang honey was able to produce results as effective as HRT, it can be used as an alternative in order to prevent bone loss with minimal side effects. However, there are few studies performed to observe the effects of Tualang honey on bone metabolism. More studies and clinical trials are required to explore the mechanism of Tualang honey on overall bone metabolism and its side effects.
